# Hospital Medical Schools and the King's Fund

**Published:** 1910-12-24

**Authors:** Savile Crossley, Frederick M. Fry

**Affiliations:** Hon. Secretaries.


### HOSPITAL MEDICAL SCHOOLS AND THE KING'S FUND.

The following letter has been addressed by the Executive Committee of King Edward's Hospital Fund for London to the Metropolitan Radical Federation, in reply to their letter of November 25 :—

King Edward's Hospital Fund for London.
7 Walbrook, E.C., December 10, 1910.

Councillor E. Garrity, M.I.I.,
Hon. Secretary,
Metropolitan Radical Federation.

Dear Sir,—Your letter of November 25 has been considered by the Executive Committee of the Fund, and we are directed to say that as you do little more than repeat in different words the several allegations made in your first communication, they must refer you to their previous reply, in which each of the charges was completely answered.

The Committee made no direct reference to your allusion to Dr. Slater's speech. But the fact that, as you have already been informed, the Fund was in correspondence with St. George's Hospital in 1909 shows that there is no ground for the suggestion that precautions were not being taken. It must, however, be evident to any fair-minded person that the Fund would not allow itself to be influenced for or against a hospital, on a question of fact, by expressions of opinion on the part of persons connected with that hospital, and the precautions referred to would have been taken even if Dr. Slater had never spoken.

The previous reply also pointed out that the decision, which you say you "learn with regret," on the subject of the payments made to medical schools before the date of Sir Edward Fry's Commission, was the decision not of the Fund, but of the Commission itself, and was contained in their report, to which, as you stated in your previous communication, your Federation "appended its cordial adhesion."

With reference to the Constitution of the Fund, the Executive Committee do not consider it within their province to enter into a discussion on a matter which was settled by Parliament little more than three years ago. They have no reason to believe that that Constitution is generally regarded as unsatisfactory, and they have already shown in this correspondence how baseless are the charges of neglect which your Federation has brought against the administration of the Fund by the governing body appointed under the Act.

With reference to your statement, which we see in the Press, that the hon. secretaries had "omitted" to publish the letter written by Sir Arthur Bigge by command of the King, we have only to say that we had not been authorised to communicate to the Press a letter addressed to your Federation, and not to us, by his Majesty's Private Secretary.

In conclusion, the Committee would repeat that the question of the financial relations between St. George's Hospital and its Medical School has been the subject of correspondence between the Fund and the hospital during 1909 and 1910, and the judgment of the Fund on the accounts published since the last grant was made will be announced in due course. In the meantime the Executive Committee, who have now twice refuted the suggestion that the Fund has already come to a decision contrary to the findings of Sir Edward Fry's Commission, or has failed to take precautions to see that its own rule based on these findings is observed, do not think it would be proper for the Fund either to forecast its decision or to discuss the question with any third party.—Yours faithfully,

(Signed) Savile Crossley,

Frederick M. Fry,

Hon. Secretaries.

